# Management of Oro-antral Communication and Fistula: Various Surgical Options

**Published:** 2017-01

**Authors:** Pulkit Khandelwal, Neha Hajira

**Affiliations:** 1 Department of Oral and Maxillofacial Surgery, Vidhyadhar Nagar, Jaipur, Rajasthan, India;; 2 Department of Prosthodontics, Khushi Dental Care and Implant Centre, Bangalore, India

**Keywords:** Management, Oro-antral, Closure, Fistula, Flap

## Abstract

Oro-antral communication and fistula can occur as a result of inadequate and improper treatment. Inadvertent communication with the maxillary sinus can occur during certain surgical procedures in the maxillary posterior region. Though, spontaneous healing may occur in defects which are smaller than 2 mm but larger communications require immediate attention and should be treated without delay, in order to avoid sinusitis and further complications leading to patient discomfort.

## INTRODUCTION

*Oro-Antral Communication*

Oro-antral communications and fistulas (OACs & OAFs) are complications frequently encountered by oral and maxillofacial surgeons. Oro-antral communication is an unnatural communication between the oral cavity and the maxillary sinus. These complications occur most commonly during extraction of upper molar and premolar teeth (48%). The major reason is the anatomic proximity or projection of the roots within the maxillary sinus.^1^Other causes of OAC/OAF include tuberosity fracture, dentoalveolar/periapical infections of molars, implant dislodgement into maxillary sinus, trauma (7.5%), presence of maxillary cysts or tumors (18.5%), osteoradionecrosis, flap necrosis, dehiscence following implant failure and sometimes as a complication of the Caldwell-Luc procedure.^1^^,^^2^

In the absence of any infection of maxillary sinus, the defects which are smaller than 2 mm can heal spontaneously following the blood clot formation and secondary healing. However, untreated larger defects can lead to development of acute sinus disease like sinusitis (50% of patients within 48 hours, 90% of patients within 2 weeks).^2^^,^^3^ Closure of this communication is very important to prevent any food or saliva accumulation. It can cause sinus contamination leading infection, impaired healing and chronic sinusitis.^4^

Wassmund reported development of sinusitis in 60% of cases by fourth day after sinus exposure while Eneroth and Martensson reported a sinusitis rate of 50% by the third day after OACs occured.^5^ Therefore, a confirmatory and early diagnosis of OACs is mandatory to permit successful closure. Also, management of oro-antral communication to promote closure should be done within 24 hours. In cases with larger oro-antral communications and in patients with history of any sinus disease, surgical closure is indicated.^2^^,^^3^

*Oro-Antral Fistula*

An oro-antral fistula (OAF) is an epithelialized pathological unnatural communication between oral cavity and maxillary sinus. It develops when the oro-antral communication fails to close spontaneously, remains patent and gets epithelialized.^5^ There is migration of oral epithelium into the defect.^1^ This epithelialization usually occurs when the perforation persists for at least 48-72 hours. Within few days, the fistula gets organized and with the epithelialization of the fistulous tract, osteitis of the surrounding bony margins, presence of foreign bodies or development of maxillary sinusitis, spontaneous healing is hampered which may result in chronic fistula formation.^1^^,^^2^ Szabo found out that 7-8 days is the average time during which an oro-antral perforation epithelialize and become a chronic fistulous tract.^6^ OAF can be further classified as alveolo-sinusal, palatal-sinusal and vestibulo-sinusal.^4^

*Diagnosis*

Patient usually complains of nasal regurgitation of liquid, altered nasal resonance, difficulty in sucking through straw, unilateral nasal discharge, bad taste in the mouth and whistling sound while speaking. Pain may be present at malar region. At later stage, there is formation of antral polyp which is visible through the defect intra-orally. However, some patients may be asymptomatic. Clinically, a large fistula is easily seen on inspection. However, diagnosis of small defect scan be made by the nose blowing test. The patient is asked to close his nostrils and blow gently down the nose with the mouth open.^2^^,^^6^^,^^7^

Presence of OAF appears as a whistling sound as air passes down the fistula into the oral cavity. It can also be seen as air bubbles, blood or mucoid secretion around the orifice. The escape of air through the nostril can be tested by placing a cotton wisp near the orifice. A mouth mirror placed at oro-antral fistula causes fogging of the mirror. Probing (the introduction of a probe into the antrum through the fistula) should never be attempted. Panoramic radiograph gives an accurate estimation of the dimension of the bony defect of the fistula and also reveals about the presence and location of dental roots or implants or any foreign body that may have been dislodged into the antrum. Computed tomography can be done to rule out the presence of maxillary sinusitis.^2^^,^^6^^,^^7^

*Perioperative Management*

Preoperatively, the affected maxillary sinus should be irrigated through the fistulous opening with normal saline followed by an iodine-containing solution diluted with normal saline (1:1; betadine) to eradicate infection. This regimen should be administered until the lavage fluid is clear and no longer contains inflammatory exudates. Numerous surgical procedures have been advocated for closure of OAC/OAF which prevents undesirable and harmful consequences of persistent OAC/OAF. These procedures may be categorised into local flaps, distant flaps and grafting. These include rotating or advancing soft tissues such as buccal flap, palatal flap, submucosal tissue, buccal fat pad and tongue flap.^2^^,^^7^

Most of these techniques mobilize and advance the resultant flap into the defect. The procedures utilizing buccal mucoperiosteal flap for closure include straight-advancement flap, rotation-advancement flap, transverse flap and sliding flap techniques. While those utilizing palatal mucoperiosteum are straight-advancement flap, rotational advancement flap, hinged flap and island flap procedures.^2^^,^^7^ Double-layer closure utilizing local tissues include the combination of inversion and rotational advancement flaps, double overlapping hinged flaps, double island flaps and superimposition of reverse palatal and buccal flaps. The most common methods used for closure of OAF are the buccal flap and the palatal pedicled flap techniques.^2^^,^^7^

*Postoperative Management*

The patients should be instructed not to eat hard food items. They should eat soft food items and drink fluid from the opposite side to avoid trauma to operated site. Strenuous physical activities which can increase the intra-sinusoidal pressure should be avoided until healing occurs. Nose blowing and sneezing with a closed mouth is prohibited for 2 weeks. Patient should open mouth while coughing or sneezing. Patients should not roll tongue over suture line or the flap for 07 days after surgery.The wound should be kept clean with warm saline mouth rinses. Use of straw or smoking is prohibited.Use of steam inhalations such as menthol or benzoin 6 hourly moistens the airway and stimulates serous gland activity preventing crusting of blood and mucous.^2^^,^^7^

All patients should receive amoxicillin plus clavulanic acid (Augmentin/Amoxyclav), 1 g twice daily, or clindamycin, 300 mg 3 times daily for at least 5 days, and a decongestant nasal drops (Otrivin 0.05%).Nasal decongestants shrink the nasal mucosa and keep the antral opening patent for drainage. Non-steroidal anti-inflammatory drugs (NSAIDS) should be prescribed for pain control.^2^^,^^7^

## DISCUSSION

Closure of OAF is a significant problem considering the undesirable consequences of sinus infection, impossibility to perform implant rehabilitation or pre-implant surgical procedures.^2 ^At the time OACs develop, if the sinus is not infected and a normal blood clot formation occurs within the socket, spontaneous closure is most likely.^5^ However, spontaneous closure is put at risk by perforations > 4 mm in diameter, socket depth >5 mm or soft tissue damage at the gingival margin.^8^

Small perforations through healthy tissues will show good prognosis for primary healing, only if the socket is blocked with a satisfactory blood clot. The blood clot formed should be protected against dislodgement. If sufficient tissues are available, primary closure should be done. Bite gauze sponge should be placed between the jaws to protect the blood clot. While discharging patient, instructions to minimize clot dislodgement should be given. Gel foam should not be inserted in the socket as it will swell up and may penetrate into sinus affecting the healing process.^6^

There are 2 basic principles that must be considered while operating for OAFs/OACs. The first is that the sinus must be free of any type of infection with adequate nasal drainage. The second is that closure must be tension free and consists of broad based, well vascularized soft tissue flap over the intact bone. Successful closure of the oro-antral fistula should be preceded by the complete elimination of any sinus pathology, the fistulous tract, sinus infection, degenerated mucosa and diseased bone.^1^^,^^9^

The success rate of immediate closure of acute oro-antral defects has been reported to be as high as 95%, but secondary closure of OAFs has low success rate, as low as 67%.^1^^,^^4^^,^^10^ The Rehrmann flap, raised by mobilizing the buccal mucosa, is most commonly used for closure of OAF.^10^

*Soft Tissue Closure: Buccal Flap*

In 1930, Axhausen first described the use of a buccal flap with a thin layer of buccinator muscle for closure of an oro-antral defect.^11^ Later, Berger advocated a buccal sliding flap technique for closure of small to medium sized (< 1 cm) fistulas which are located either laterally or at center of the alveolar process.^12^ Krompotie and Bagatin described immediate closure of an oro-antral communication and fistula by rotating gingiva-vestibular flap.^13^ This technique is a modification of vestibular flap which prevent lowering of the vestibular sulcus, which occurs normally while using vestibular flaps.^13^

After excising the epithelialized margins, two vertical release incisions are given to develop a flap with adequate dimensions for closure of the oro-antral defect. Epithelial lining of the palatal mucosa behind the communication is also excised. This trapezoidal flap consists of both epithelium and connective tissue. The flap is then placed over the defect and sutured using horizontal mattress sutures from the buccal mucosa to the palatal mucosa. The advantage of the buccal flap technique is that this method can be used when the alveolar ridge height is very low and the fistula is located in a more mesial area. However, the loss of the vestibular depth represents a major disadvantage requiring an additional vestibuloplasty in patients wearing removable dentures.^4^

*Palatal Flap*

Ashley was first to describe a technique for closure of oro-antral fistulas using a palatal full thickness flap. In 1980, Ehrl employed this technique with large fistulas of 1cm in diameter. Yamazaki further modified this technique by adding a flap of mucosa to the connective tissue island which covered the raw area of palate. The island flap retains excellent mobility and there is no damage or bunching of the mucosa of the hard palate and recipient site. This technique allows denture wearing a short time after the wound healing. The bony surface is preserved as the periosteum remains intact.^4^

The epithelium is excised from its edges. Then the palatal fibro-mucosa is incised so as to raise a flap having a posterior base, which is supplied by the greater palatine artery. The anterior extension of the flap must be sufficiently wide to exceed the diameter of the bony defect and sufficiently long to allow lateral rotation. Tension free suturing should be done.^14^ However, the palatal flap is feasible only in cases of closing fistulas in the premolar region. In molar region, excessive tension may cause ischemia of the flap due to occlusion of greater palatine artery. Advantages of the palatal flap include high vascularity, adequate thickness and quality of tissue. The most serious disadvantage is the flap necrosis which can occur due to excessive rotation of the flap. Other disadvantages of this technique include exposed bony surface, pain and surface irregularities may develop due to secondary epithelialization post operatively.^4^

*Buccal Pad of Flat Flap (BFP)*

According to Egyedi and Hao, BFP flap is a satisfactory method to close the oro-antral defects. Rapid epithelialization of the uncovered fat is a peculiar feature of the BFP flap stalk. An incision is made in posterior mucosa in the area of the zygomatic buttress, followed by a periosteal incision. Fascia enveloping the buccal pad of fat is incised then. A gentle dissection with fine curved artery forceps is done to expose the yellowish-colored buccal fat. The pedicled buccal fat pad flap is used most commonly for the closure of the OAF. The reason for favourability of using BFP is anatomically favourable location; ease of harvesting and minimal dissection required to harvest and to mobilize the flap. The advantages of this technique include good epithelialization and a high rate of success. The disadvantages include decrease in the vestibularheight.^4^

*Alternative Approaches for the Closure of OAC*

Ziemba described a two flap technique for the closure of oro-antral fistula. Advantage of using two flaps over one flap is that it provides stable epithelial covering to both the superior and inferior surfaces of the repaired defect. This reduces the incidence of contracture and postoperative infection as well. This reduces the chances of breakdown of wound and recurrence of the defect.^15^ A double layer flap closure method was described by Batra *et al.*, to repair the OAF. Buccal Fat Pad (BFP) covered the defect with an overlying closure with a layer of buccal mucosal flap. Hassan *et al.* used combined palatal–buccal flap technique for the late repair of small to medium-sized OAF.^1^

Bone autografts have been recommended in the literature for closing OAFs.^16^ Autografts harvested from the extraction socket, intra-oral sites like anterior mandible, or distant sites like iliac crest have been used for repairing the bony defect in maxilla.^5^Interseptal alveoloplasty offers the advantage of facilitating spontaneous postoperative healing, supported by the bony base. However, this technique is of limited usefulness for closure because it requires a residual alveolar process of sufficient height and width and an intact buccal cortical layer.^5^^,^^17^ Sinus closure with bone graft harvested from the iliac crest was first reported by Proctor. It should, however, be indicated for large defects because of the known donor site morbidity inherent to it.^16^ Haas *et al.* proposed press-fitting monocortical block grafts harvested intra-orally for closing OAFs. They successfully treated many patients with this technique.^3^

Auricular cartilage can be used for the closure of oro-antral fistulas. Auricular cartilage is biocompatible, highly resistant to infection, easy to harvest and manipulate, non-resorbable and cost-effective. This graft does not require vascularisation for the integration to the recipient site. This characteristic feature decreases the failure rate of the graft. There is no scar or defect formation at the donor site. Auricular cartilage graft act as a barrier between the sinus membrane and the oral mucosa which allows successful healing. The only requirement for this technique is that the auricular graft must be supported by primary closure.^18^

For maxillary sinus lift surgery, the sinus membrane should be intact without any inflammation. However, chronic OAFs usually lead to severe chronic inflammatory thickening of the sinus membrane and hence, sinus lifting cannot be proceeded. Soft tissue closure of OAFs without bony reconstruction before implant surgery carries a major risk of injury to oral mucosa and sinus membrane during sinus augmentation. Sinus closure with autogenous grafts paves the way for subsequent conventional sinus lifting. The regeneration of the osseous support of soft tissue flaps is an alternative and effective treatment option for closure of OAFs, especially in cases where secondary closure is required. This technique facilitates subsequent conventional sinus lifting and also preserves the teeth adjacent to OAFs.^18^

Yoshimasa *et al.* advocated 3^rd^ molar transplantation as a suitable technique for closure of OAC without the need for further prosthodontic treatment in case of single missing tooth in the region**.**^19^ Ogunsalu described sandwich technique for the closure of oro-antral defects, in which both hard tissue (bone) as well as soft tissue closure was achieved. In this technique, a bone grafting material was sandwiched between two sheaths of a bio-resorbable membrane for the hard tissue closure of oro-antral defect. There was excellent bony regeneration which allowed placement of an endosseous implant.^19^ Scattarella *et al.* described guided-tissue regeneration (GTR) technique in which an autologous bone graft was integrated with xenologous particulate bone graft, and was covered with a non-reabsorbable, expandable GTR membrane. This method helped in the reconstruction of the lost bone tissue as well as prevented epithelial migration to the grafted area.^2^

Complete closure of oro-antral defect can be achieved by using a single application of lyophillized fibrin seal. The sealant mixture can be placed above the floor of the antrum to protect the clot from air flow.^20^ In 1992, Zide and Karas used nonporous hydroxylapatite (HA) blocks to close chronic fistula and OAF. A nonporous HA block is cut to fit the bony defect and secured to alveolar bone using a 26-gauge wire. Advantages include the ability to have a press-fit graft closure, no morbidity associated with a second-site surgery and the exposure of the block in case if soft tissue closure cannot be achieved. A resorbable collagen membrane can also be placed over the OAC and secured with resorbable pins.^21^

The membrane protects the blood clot and help in organization of clot so that blood clot gets replaced by bone and epithelium on the oral surface. Use of gold foil or gold plate has been reported in many studies for the closure of OACs. The gold foil is burnished into defect with its edges around on a healthy bone, thus acting as a protective barrier for overgrowing sinus mucosa. Aluminum foil found in dental film packages can be used as an alternative to gold foil, to cover the OAC. Laser light in low doses has also been used successfully for the closure of OACs. Patients are exposed to a biostimulative laser of 30 mW power for 10.5 minutes for 4 consecutive days.^21^

*Failure*

Any surgical procedure can have chances of failure. The most common reasons leading to failure after closure of oro-antral defects include^6^: inadequate preoperative irrigation and antibiotic therapy for any existing sinus infection or disease, excessive tension on the flap impairing blood supply for healing, inadequate excision of epithelialized margins and inadequate trimming of bony margins prior to closure or post-operative instructions not given properly or negligence on part of patient to follow the instructions.^6^

## CONCLUSION

Repairing oro-antral defects like OAC/OAF is one of the most challenging and difficult problems in the field of oral and maxillofacial surgery. In selecting the surgical approach to close an oro-antral fistula, different criteria must be taken into consideration, like location of defect, size of defect, height of the alveolar ridge, vestibular depth, persistence of defect, sinus inflammation or infection and general health of patient. OAC/OAF should be managed promptly by creating a barrier between oral cavity and maxillary sinus to prevent maxillary sinusitis. Treatment modalities to repair the oro-antral defects include local or free soft tissue flaps, with or without autografts or alloplastic materials. The buccal flap is suitable for closure of small and mesial fistulas; the palatal flap is a feasible option for repairing OACs, more likely for defects in the premolar area. The BFP is suitable for the closure of large posterior OAC/OAFs.
